# Editorial: Autonomic Nervous System and Cardiovascular Diseases: From Brain to Heart

**DOI:** 10.3389/fphys.2022.884832

**Published:** 2022-04-29

**Authors:** Yueyi Wang, Liping Zhou, Hong Jiang, Lilei Yu

**Affiliations:** ^1^ Department of Cardiology, Renmin Hospital of Wuhan University, Wuhan, China; ^2^ Cardiac Autonomic Nervous Research Center of Wuhan University, Wuhan, China; ^3^ Cardiovascular Research Institute, Wuhan University, Wuhan, China; ^4^ Hubei Key Laboratory of Cardiology, Wuhan, China

**Keywords:** autonomic nervous system, sympathetic, parasympathetic, brain-heart connection, cardiovascular disease

The autonomic nervous system connects the control from brain to heart through the sympathetic and parasympathetic branches. Central nervous system diseases can induce cardiovascular diseases mediated by autonomic nervous system. For instance, patients with Parkinson’s disease present autonomic dysfunction and α-synuclein deposition in cardiovascular system leading to catecholaminergic abnormalities and cardiac denervation, because of the high density of sympathetic innervation in the heart (Sharabi et al., 2021). Meanwhile, the brain-heart interaction is involved in the pathophysiology of cardiovascular diseases through autonomic nervous system, as patients with Takotsubo syndrome display deficient autonomic-limbic integration and fewer functionally connected parasympathetic- and sympathetic-subnetworks (Templin et al., 2019). All these findings highlight the autonomic nervous system as an essential target for prevention and control of the pathological process of cardiac diseases, and for further clinical transformation (Yu et al., 2010; Yu et al., 2017).

This research topic aimed to explore recent developments in this area focused on 1) the role of autonomic nervous system in cardiovascular diseases, 2) the role of autonomic nervous system in central nervous system diseases, and 3) the autonomic nervous system interaction between heart and brain.

The function of cardiac autonomic nervous system (CANS) is under influence of multiple factors, including pathophysiological processes. Hooper et al. showed that irritant-evoked pulmonary-cardiac reflexes were remodeled in spontaneously hypertensive (SH) rats. Inhalation of the selective transient receptor potential ankyrin 1 agonist allyl isothiocyanate evoked pulmonary-cardiac reflex, leading to morphological change in P waves in SH rats. Inhibition of either parasympathetic or sympathetic components of the pulmonary-cardiac reflex attenuated these effects. This study provides novel evidence for altered irritant-evoked pulmonary-cardiac reflex for AF initiation in SH rats, that deepens our understanding of autonomic mechanisms underlying cardiovascular diseases.

Kidney plays an important role in sympathetic activation and the pathogenesis of hypertension, and regulation of the renal afferents influences the sympathetic activity and blood pressure. Ye et al. demonstrated the contribution of renal afferents and the induced excitatory renal reflex (ERR) in the development of hypertension in SH rats. The study showed that ERR was enhanced in the early stage of hypertension, but attenuated in the later stage in the SHR, indicating the involvement of abnormal ERR in sympathetic activation and the development of hypertension. As the renal sympathetic denervation therapy gains extensive attention, this study proposed that selective renal afferent or efferent denervation may produce distinct output in hypertension, especially at the early stage.

The stellate ganglion (SG) is an autonomic nervous ganglion that provides sympathetic outflow and plays an integrative role in regulating cardiovascular function. Myocardial infarction (MI) results in neural remodeling in the SG, however, the expression and function of the proteins in SG tissue after MI remained unclear. Cheng et al. explored the expression characteristics of proteins in rabbit SG by the tandem mass tags quantitative proteomic sequencing, and found 383 differentially expressed proteins (DEPs) including 143 upregulated and 240 downregulated proteins. This study provides beneficial evidence for further studies on the SG in understanding the pathological process and developing therapeutic treatment of MI.

Along with the identification and investigation of the autonomic mechanisms in cardiovascular disease, major focus was put on the left-sided cardiac pathology and in animals. However, the structure, function and autonomic innervation of the right ventricle are distinct from the left. Zandstra et al. reviewed the asymmetry and regional differences with distinct anatomical, functional and molecular characteristics of the CANS in humans. Based on this review, further researches are needed to investigate the different autonomic innervation of the right ventricle and design accurate treatment in accordance with cardiac autonomic asymmetry and heterogeneity.

Cardiac autonomic imbalance is observed after acute ischemic stroke (AIS), and heart rate variability (HRV) can be used to predict clinical outcomes and neurological function. However, are other noninvasively measured cardiac parameters involved and associated with neurological improvement in AIS? Joseph Miller et al. conducted a pilot prospective observational study in AIS patients, and found that the cardiac stroke volume index (cSVI) and mean arterial blood pressure (MAP) were associated with 24-h neurological improvement. Of which, cSVI showed a linear correlation with NIH stroke scale improvement. This study proposed cSVI as a unique cardiac parameter in association with 24-h neurological outcomes in AIS patients, which provides valuable evidences for possible therapeutic and prognostic application.

Hydrogen sulfide (H_2_S), as a vital endogenous gasotransmitter, is involved in a wide range of physiological and pathological processes. Basak Donertas Ayaz et al. studied the H_2_S donor NaHS in a rat model of hypertension. They found that intracerebroventricular infusion of NaHS attenuated angiotensin II induced hypertension, autonomic dysfunction and microglia activation in paraventricular nucleus (PVN). Their results suggest an independent role of central H_2_S from circulating H_2_S in treatment of hypertension, and the mediated neuromodulating and neuroimmune pathways contributing H_2_S a potentially beneficial autonomic and anti-hypertensive target.

Epilepsy is a disorder of central nervous system characterized by repeated seizures, leading to impaired CNAS function. However, there are few researches studied the CANS function in pre-school children with epilepsy. Yang et al. found that the measurements of heart rate variability (HRV), multiscale entropy (MSE) and Kurths-Wessel symbolization entropy (KWSE) were significantly lower in pre-school pediatric intractable epilepsy (PIE) patients, indicating the imbalance of CANS in both sympathetic and vagal tone. Meanwhile, an accurate prediction of PIE *via* the combination of HRV, MSE and KWSE was proposed based on noninvasive ECG. This study provides important implication in health control in pre-school PIE children.

Event-related potentials (ERPs) are commonly used to assess motor or cognitive events. But no study reported if other factors, such as changes in heartbeat-evoked potentials (HEPs), could influence ERPs. Park et al. revealed the effects of HEPs on the performance of the mental workload (MWL) classification based on ERPs. With a mental arithmetic task to distinguish low- and high- MWL, they found that HEPs affected the ERPs resulting in a decrease in the performance of MWL classification. HEPs reflected a synchronization in the communication between brain and heart, indicating that the cardiac activity is needed to be considered to obtain a clear and pure ERP response. This study suggested an accurate strategy to improve brain activity classification and provide a better application of ERPs to various fields.

Both sympathetic hyperactivation and baroreflex dysfunction are typical characteristics in heart failure patients with reduced ejection fraction (HFrEF), but it’s unclear between the phasic activity of sympathetic nerve bursts and the baroreflex dysfunction. Toschi-Dias et al. investigated the rhythm of the muscle sympathetic nerve activity (MSNA) in HFrEF patients. They found that the oscillatory pattern of MSNA was directly associated with the gain and coupling of the sympathetic baroreflex function, and inversely associated with MSNA burst frequency. This study extended our knowledge about the oscillatory pattern of MSNA on the functional capacity and clinical condition of HFrEF patients.

Moyamoya disease is a rare cerebrovascular disease resulting in ischemic or hemorrhagic stroke, characterized by progressive stenosis of the intracranial internal carotid arteries and their proximal branches. Zou et al. presented a rare case of a postpartum woman with moyamoya disease, observed atypical posterior reversible encephalopathy syndrome (PRES). Based on this report, PRES can occur in patients with moyamoya disease and should be considered for the differential diagnosis of cerebral infarcts and hemorrhage in postpartum woman, providing both pathophysiological and clinical significance.

In conclusion, the present research topic collected some interesting papers and revealed better understanding of the autonomic nervous system interaction between heart and brain. We hope further researches about this topic will be continued, and will contribute to the clinical transformation of novel neuromodulate strategies.
